# A novel quantitative electroencephalography subtype with high alpha power in ADHD: ADHD or misdiagnosed ADHD?

**DOI:** 10.1371/journal.pone.0242566

**Published:** 2020-11-17

**Authors:** Jun Byeon, Tae Young Choi, Geun Hui Won, Jaewon Lee, Jun Won Kim

**Affiliations:** 1 Department of Psychiatry, Catholic University of Daegu School of Medicine, Daegu, Republic of Korea; 2 Department of Psychiatry, Easybrain Center, Seoul, Republic of Korea; Hunan Normal University, CHINA

## Abstract

This study investigated quantitative electroencephalography (QEEG) subtypes as auxiliary tools to assess Attention Deficit Hyperactivity Disorder (ADHD). A total of 74 subjects (58 male and 16 female) were assessed using the Korean version of the Diagnostic Interview Schedule for Children Version IV and were assigned to one of three groups: ADHD, ADHD-Not Otherwise specified (NOS), and Neurotypical (NT). We measured absolute and relative EEG power in 19 channels and conducted an auditory continuous performance test. We analyzed QEEG according to the frequency range: delta (1–4 Hz), theta (4–8 Hz), slow alpha (8–10 Hz), fast alpha (10–13.5 Hz), and beta (13.5–30 Hz). The subjects were then grouped by Ward’s method of cluster analysis using the squared Euclidian distance to measure dissimilarities. We discovered four QEEG clusters, which were characterized by: (a) elevated delta power with less theta activity, (b) elevated slow alpha relative power, (c) elevated theta with deficiencies of alpha and beta relative power, and (d) elevated fast alpha and beta absolute power. The largest proportion of participants in clusters (a) and (c) were from the ADHD group (48% and 47%, respectively). Conversely, group (b) mostly consisted of the participants from the NOS group (59%), while group (d) had the largest proportion of participants from the NT group (62%). These results indicate that children with ADHD does not neurophysiologically constitute a homogenous group. We also identified a new subtype with increased alpha power in addition to those commonly reported in ADHD. Given the QEEG characteristics with increased alpha power, we should consider the possibility that this subtype may be caused by childhood depression. In conclusion, we believe that these QEEG subtypes of ADHD are expected to provide valuable information for accurately diagnosing ADHD.

## 1. Introduction

Attention deficit hyperactivity disorder (ADHD) is characterized by three major symptoms: attention deficits, hyperactivity, and impulsivity [1]. The prevalence of ADHD ranges from 2 to 18% in those between the ages of 6–17 years in the USA [2], and a recent meta-analysis found the ADHD prevalence to be 7.2% worldwide [3]. Among the 13.5% of children and adolescents with mental disorders worldwide, ADHD is one of the most common mental disorders [4].

While ADHD has a very high prevalence among children and adolescents, it is difficult to clinically diagnose precisely. ADHD has many comorbidities such as mood disorders, oppositional defiant disorder, conduct disorder, anxiety disorders, and sleep disorders, making ADHD diagnosis difficult [5]. Furthermore, the Diagnostic and Statistical Manual of Mental Disorders-5 (DSM-5) is commonly used to diagnose ADHD categorically. However, its diagnostic accuracy is controversial, whereby ADHD is commonly over-diagnosed in patients with different diseases and the normal variant is diagnosed with ADHD [6]. Therefore, many attempts have been made to improve the accuracy of ADHD diagnosis, especially around the development of evidence-based diagnostic methods and treatments to quantify changes in the brain and use them as diagnostic criteria. Currently, functional magnetic resonance imaging (f-MRI), magnetic resonance imaging (MRI) [7,8], polymerase chain reaction (PCR)—used for analyzing single nucleotide polymorphisms including rs5320, rs2075654, rs1079596, and others in genes encoding proteins such as dopamine β-hydroxylase and dopamine receptors [9]—and quantitative electroencephalography (QEEG) [10–12] have been implemented as auxiliary tools to assess ADHD. Among these methods, QEEG–which measures brain function by obtaining data from the electrophysiological activity of the brain–is expected to greatly help diagnose, understand, and determine the appropriate treatment for ADHD [13]. In addition, QEEG is especially advantageous for pediatric patients as it is relatively cost-effective, quicker, and non-invasive compared to other diagnostic tools that may involve needles or radiation.

Currently, there are three subtypes of ADHD classified using QEEG. The first is the maturational lag subtype characterized by increased slow wave and decreased fast wave activity [14]; EEG activity changes from a slow wave dominance to fast wave dominance between childhood and adolescence [15]. The characteristic of this subtype is that if the age of the QEEG comparator is lowered, the abnormal pattern is eliminated and normalized. The second subtype is hypoarousal, marked by increases in theta wave and decreases in beta wave activity [14]. This pattern highlights thalamo-cortical dysrhythmia, known to cause various mental disorders [16]. Third is the hyperarousal subtype, which shows greatly increases the activity of the cortex characterized by increased beta wave activity [14]. Clinically, it is also the type that is least responsive to traditional ADHD medication [17]. The hypoarousal subtype is the most commonly identified ADHD subtype based on QEEG results; however, as mentioned, its characteristics are also observed in other disorders [18]. Nevertheless, QEEG results in ADHD are known to be more consistent than those in other diseases [19].

Research on the diagnostic accuracy of resting QEEG in ADHD has been conducted previously [10–12]. However, there is a lack of data on the ADHD subtypes and diagnostic QEEG values for these subtypes. Accordingly, we aimed to determine the diagnostic benefits of resting QEEG by analyzing resting QEEG in ADHD through cluster analysis to determine the characteristics of each group.

## 2. Material and methods

### 2.1. Subjects

Individuals who visited the child and adolescent psychiatric clinic at Daegu Catholic University Hospital from 2018 to 2020 were considered for the study. Participants between 7 and 12 years of age diagnosed with ADHD according to the DSM-5 criteria were included in the study. The ADHD diagnosis was based on a Korean version of the Diagnostic Interview Schedule for Children Version IV (DISC-IV), which is a structured interview tool, and these diagnoses were confirmed by multiple child and adolescent psychiatrists. If participants did not meet the ADHD diagnostic criteria of DSM-IV and DISC-IV, they were assigned to the Neurotypical (NT) group. Based on the results of the DISC-IV test, participants were assigned to the ADHD or Non-Other Specified (NOS) group. Patients who met the diagnostic criteria of ADHD in DSM-IV, but whose score did not exceed six, and had a score of more than three in the attention deficit or hyperactivity/impactivity scale of DISC-IV were included in the NOS group. Children with a history of brain damage, neurological disorders, genetic disorders, substance dependence, epilepsy, or any other mental disorder were excluded from participation. Children with an IQ of 70 or lower according to the Korean-Wechsler Intelligence Scale for Children (Fourth Edition) or who were receiving drug treatment were also excluded from this study. Based on the inclusion and exclusion criteria, 74 subjects (58 male and 16 female) were enrolled in this study. Detailed information regarding the study was provided to the parents and children. Written consent for the medical use of the test results and participation of the children in this study were obtained from all of the participants’ parents. In addition, after receiving a detailed explanation of the study, all of children participated voluntarily and provided written consent for participation. This study was approved by the Institutional Review Board (IRB) of the Daegu Catholic University Medical Center (DCUMC IRB approval No. CR-18-096) and was performed in accordance with the Declaration of Helsinki (World Medical Association: Ethical Principles for Medical Research Involving Human Subjects, 1964).

### 2.2. Korean version of Diagnostic Interview Schedule for Children Version IV

The DISC-IV is a structured diagnostic tool that was developed for use in epidemiological studies in children and adolescents. Using DISC-IV, the presence of nine symptoms of attention/concentration issues and nine symptoms of hyperactivity–impulsivity over the past 6 months can be assessed. When symptoms were noted, detailed questions, such as whether the symptoms were observed at home or school, were asked. The DISC-IV was revised in 2000 by the U.S. National Institute of Mental Health. The present study used the Korean version of the DISC-IV, which was translated into Korean in 2007 and subsequently underwent reliability(κ = 0.67) and validity(κ = 0.65~1.00) verification [20].

### 2.3. Korean ADHD rating scale (KARS)

For screening purposes, the 18-item Korean version of the ADHD Rating Scale was used to assess ADHD behavior [21]. The KARS is a standardized screening tool for ADHD in Korean children and rating scale completed by the parents and its reliability and validity is well established; Cronbach’s alpha index for that study was 0.85 and that for our study was 0.93 [21].

### 2.4. Continuous Performance Test (CPT)

For screening purposes, the CPT was used to assess the level of functioning of the attention/arousal system. We used the Intermediate Visual and Auditory Continuous Performance Test (IVA CPT, BrainTrain, Inc. Richmond, VA, USA. www.braintrain.com) to obtain behavioral measures of attention. The IVA CPT results yielded standardized scores of attention and the Response Control Quotient for each visual and auditory stimulus based on normative data. Cronbach’s alpha index of that study was 0.72 and that of our study was 0.7 [22].

### 2.5. EEG recording and pre-processing

The EEG recordings were performed using a SynAmps2 direct-current (DC) amplifier and a 10–20 layout 64-channel Quick-Cap electrode-placement system (Neuroscan Inc., NC, USA). The EEG data were digitally recorded from 19 gold cup electrodes placed according to the international 10–20 system (Fp1, Fp2, F7, F3, Fz, F4, F8, T7, C3, Cz, C4, T8, P7, P3, Pz, P4, P8, O1, O2). The impedances were maintained below 5 kΩ, and the sampling rate was 1000 Hz. We used the linked mastoid reference and two additional bipolar electrodes to measure horizontal and vertical eye movements. During the recording, each participant laid in a dimly lit, electrically shielded, sound-attenuated room. Resting EEG recordings were recorded after three minutes with the participants’ eyes closed.

We used MATLAB 7.0.1 (Math Works, Natick, MA, USA) and the EEGLAB toolbox [23] to pre-process and analyze the EEG recordings. First, the EEG data were down-sampled to 250 Hz. Next, the EEG data were detrended and mean-subtracted to remove the DC component. A 1-Hz high-pass filter and a 60-Hz notch filter were applied to remove eye and electrical noise. Next, independent component analysis (ICA) was performed to remove the well-defined sources of artifacts. ICA has been shown to reliably isolate artifacts caused by eye and muscle movements and heart noise [24]. Finally, clinical psychiatrists and EEG experts visually inspected the corrected EEGs. For the analysis, we selected more than two minutes of artifact-free EEG readings from the three-minute recordings.

### 2.6. EEG analysis

Five frequency bands were defined for further analysis: delta (1–4 Hz), theta (4–8 Hz), slow alpha (8–10 Hz), fast alpha (10–13.5 Hz), and beta (12–30 Hz). We investigated the power spectra of the EEG data for each subject using the short-time Fourier transform ‘spectrogram.m’ function from the Signal Processing Toolbox in MATLAB. Time windows of 1,000 ms with an 800 ms overlap and Hamming window were used for the spectral analysis. Outliers that were far from the spectral value distribution of each frequency band at the 0.05 significance level were removed. Finally, the absolute powers were averaged over all the time windows and frequency bands for further analysis. The data from participants were converted to Z scores based on the means and standard deviations of the NeuroGuide normative database [25]. The Z scores allowed for comparable estimates of excesses or deficiencies of power for each frequency band at each electrode, for each individual compared to normative database.

### 2.7. Statistical analysis

The MATLAB 7.0.1 Statistical Toolbox was used for the statistical analyses. All of the values are expressed as means and the standard deviations. First, an analysis of variance (ANOVA) was conducted on the QEEG results for the ADHD, ADHD NOS, and NT groups. Second, we used the statistical function named 'classify.m' in MATLAB to implement the classifiers of linear discriminant analysis (LDA). LDA is used to determine the linear combination of features that can better separate two or more classes [26]. LDA entails a statistical approach to reduce the dimensionality of data, by calculating the optimal projection, in order to minimize the distance within the classes and maximize the distance between classes [27]. Although its limitation includes the determination of the boundaries between the different classes with a straight line, its advantages include the simplicity of implementation, where a linear combination of features is used to separate classes of samples [28]. The variables used in the discriminant analysis included the absolute power and relative power of 19 electrodes in each of the five frequency bands. Discriminant function analysis was performed on the subject clusters identified in the cluster analysis to determine the level of correct classification of the subjects based on the EEG data. The fitting function estimates the parameters of the Gaussian distribution for each class to train a classifier, and the trained classifier determines the class with the smallest misclassification cost to predict the classes of new data. To improve the clarity of the results, topographical plots of the results of the statistical comparisons to normative values (z-scores) using Neuroguide software (Applied Neuroscience, Inc.) are presented.

## 3. Results

### 3.1. Demographic characteristics

There were 74 children (58 males, 16 females) included in the analysis. There was no significant difference between the mean ages of the males and females (t = 0.645, p = 0.521), at 8.9±1.2 years and 8.7±1.2 years, respectively. The mean KARS score for all subjects was 29.47±12.57 for males and 23.19±14.40 for females, with no statistically significant difference between males and females (t = 1.714, p = 0.091). Based on the DISC-IV results, we identified the differences between the groups by categorizing them into three groups: 27 participants (6 females) in the ADHD group, 32 participants (6 females) in the ADHD Not Otherwise Specified (NOS) group, and 15 participants (4 females) in the Neurotypical (NT) group.

### 3.2. Comparisons between groups: QEEG

Using ANOVA, the mean-to-mean difference between the three groups was investigated with respect to the absolute and relative power of the delta, theta, slow alpha, fast alpha and beta waves. Among the waves, the fast-alpha absolute power (F = 4.60, η2 = 0.114, 1-β = 0.761, p = 0.013) and beta absolute power (F = 4.29, η2 = 0.107, 1-β = 0.730, p = 0.017) showed a difference in meaningful scores between the three groups. Following the Scheffe post-hoc test, the ADHD and NOS groups were found to be significantly lower in fast alpha absolute power and beta absolute power than NT.

### 3.3. Cluster analysis and differences in topographical model

**Fig 1:**
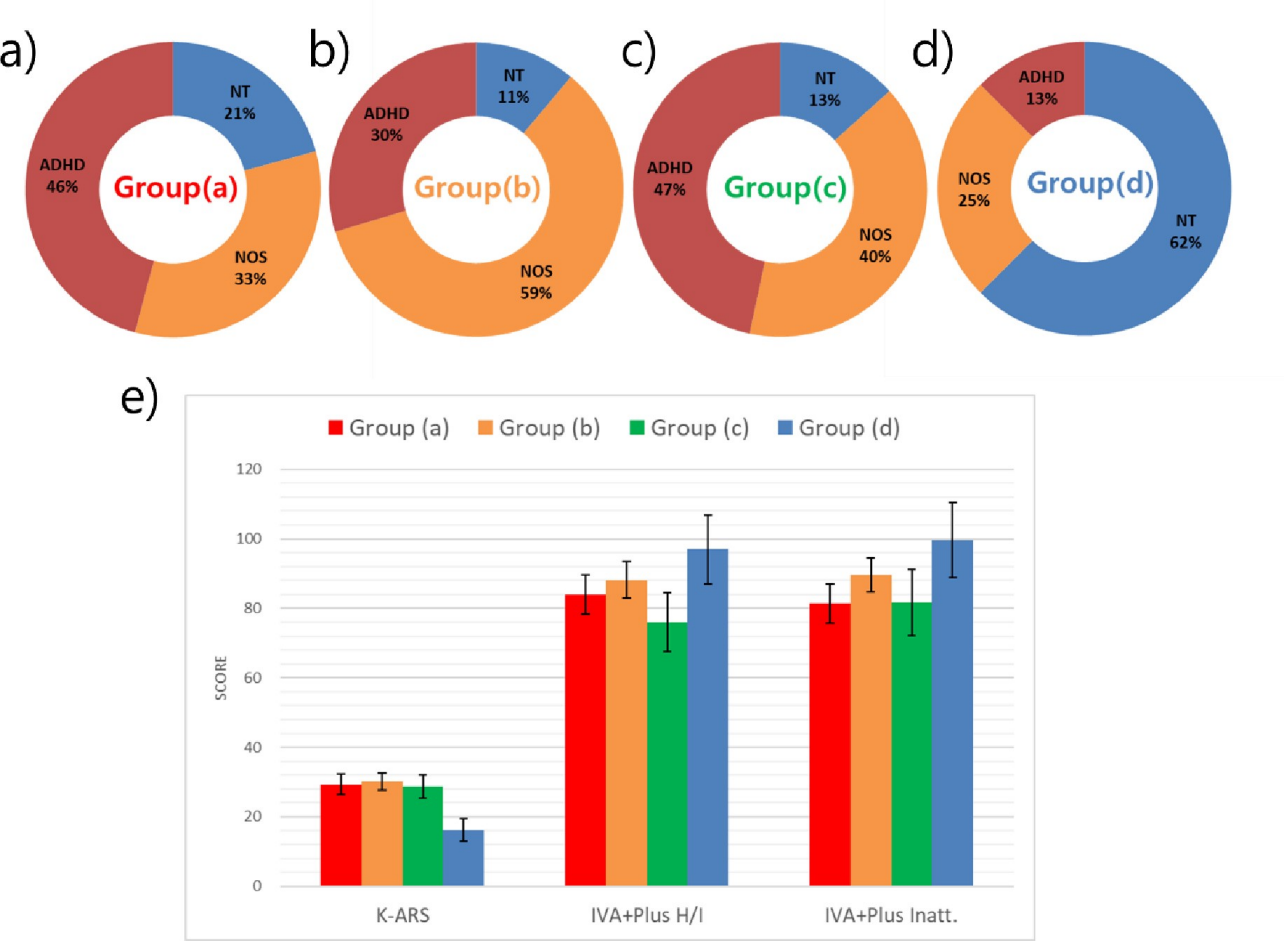
Characteristics of the four subtypes divided by cluster analysis of QEEG. Abbreviations: QEEG, Quantitative Electroencephalography; ADHD, Attention-Deficit Hyperactivity Disorder; NT, Neurotypical; NOS, ADHD Not Otherwise Specified; K-ARS, Korean ADHD rating scale, IVA+Plus, Integrated visual and auditory test; H/I, Hyperactivity/Impulsivity; Inatt., Inattention; error-bar means standard error.

Using cluster analysis, the QEEG results were divided into groups (a) to (d). The percentages of NT, NOS, and ADHD groups in each group were obtained and plotted on a circular graph (Fig 1A–1D). In addition, the mean and variance of the group's KARS and IVA+Plus scores were obtained and plotted in a bar graph (Fig 1E). When comparing each group, groups (a) and (c) included the highest proportion of participants from the ADHD group (46% and 47%, respectively) (Fig 1A and 1C), group (b) included the highest proportion of participants from the NOS group (59%) (Fig 1B), and group (d) included the highest proportion of participants from the NT group (62%) (Fig 1D).

**Fig 2:**
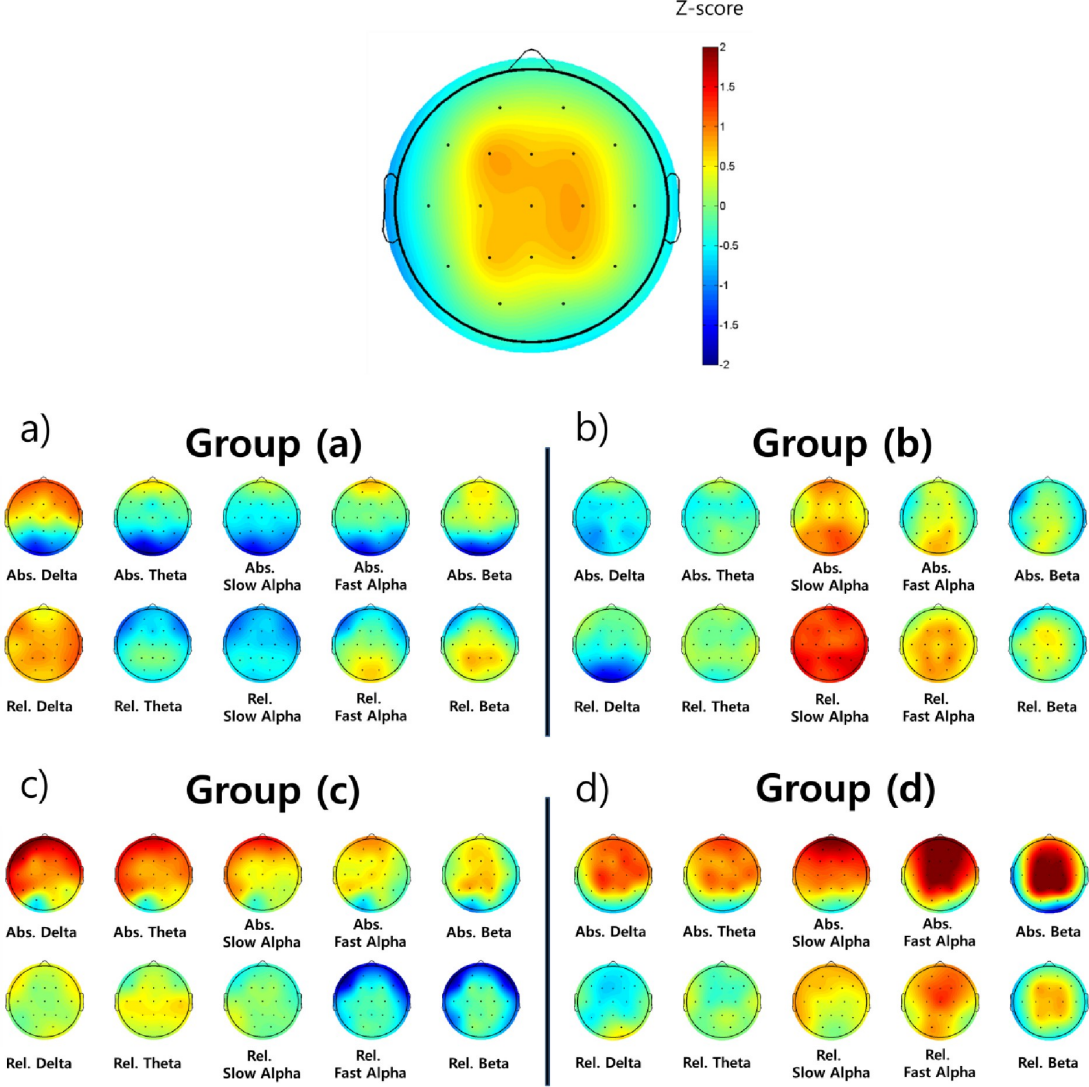
The difference in topography between the 4 subtypes divided by cluster analysis of QEEG. Abbreviations: QEEG, Quantitative Electroencephalography; Abs., Absolute; Rel., Relative.

The topography of groups (a)-(d) divided through the cluster analysis was implemented through EEGLAB (Fig 2). Groups (a) and (c) with many ADHD participants had different characteristics. Specifically, group (a) had elevated delta power with less theta activity (Fig 2A), group (c) had elevated theta with deficiencies in alpha and beta relative power (Fig 2C). Group (b) with many NOS had elevated slow alpha relative power (Fig 2B), and group (d) with many NT showed elevated fast alpha and beta absolute power, but relative power appeared relatively similar compared to the other groups (Fig 2D).

## 4. Discussion

The present study classified ADHD children into NT, NOS, and ADHD groups using a structured interview (DISC-IV), and the results of their attention test and QEEG characteristics were assessed for each group. We also divided participants into four groups through cluster analysis of the QEEG results, determined the distribution of the ADHD, NOS, and NT group, and constructed topographical plot to identify the characteristics of the QEEG results according to group.

The results showed that the demographic characteristics of the subjects did not differ in terms of age and gender. A gender ratio of 3:1 favoring males was found, similar to what has been observed in previous studies [2]. Using cluster analysis of the QEEG results, participants were divided into four. Group (a) with 46% of participants from the ADHD group had elevated delta power and low theta wave activity (Fig 2A). This result is similar to the maturation lag type [14]. As mentioned earlier, EEG signals change from slow wave as the dominant form to the dominant form of becoming fast waves when transitioning from childhood to adolescence [15]. This type of ADHD is thought to attenuate brain development compared to their age-matched counterparts [29]; the notion that ADHD symptoms tend to improve with age, and up to 80% of children tend to deviate from ADHD at adulthood supports this hypothesis [30]. In addition, cortico-striatal regions in those with ADHD identified using MRI, known to develop during late adolescence, are smaller than those in individuals in Neurotypical groups [31,32], and the lack of activity also supports this hypothesis [33]. This tendency is also shown in the CPT. Cognitive performance measured in ADHD with the CPT is similar to normal groups 1 to 3 years younger than those with ADHD [34].

Group (c) included 47% of ADHD groups and had elevated theta absolute power and low fast alpha and beta relative power (Fig 2C). This result aligns with the hypoarousal type [14]. EEG findings showing a decrease in beta waves, which reflects the pathology of ADHD in that this indicates a decrease in cortical activity [35]. A rise in theta/beta ratio (TBR), as observed in group (c), is usually observed in ADHD. The TBR can be used to distinguish between the NT and ADHD groups with 86–90% sensitivity and 94–98% specificity, and is reported as the most accurate diagnostic marker among QEEG parameters [36,37].

Group (b), which comprised of 59% of participants from the NOS group, was characterized by the elevated alpha waves. Alpha waves are functionally associated with arousal [38], and elevated alpha waves are consistent findings in depressions [39,40]. In childhood depression, the disorder can present as depressive mood, anxiety, and ADHD symptoms such as attention-deficit. These symptoms also are included in the ADHD diagnostic criteria and are difficult to distinguish between childhood depression and ADHD in clinical situations [41]. Considering these characteristics and the fact that this group mostly consisted of participants from the NOS group, patients separated in group (b) have two potential diagnoses. First, it is possible that attention deficit and concentration impairment occurred due to childhood depression [42]. Second, there is a possibility that patients have both childhood depression and ADHD [43]. Therefore, the possibility of depression may be better to explore if the QEEG appears in group (b). In this study, the score on the depression scale was expected to not show significant differences from the other groups because there were not enough subjects. Further studies will be required to explore depression symptoms and verify the hypothesis above.

Lastly, group (d) included 62% of participants from the NT, and fast alpha and beta wave absolute power were elevated, but relative power was relatively equal compared to the other groups. These elevations in absolute power may be an error of the measurement tool employed. For EEG measurements, it is important to control for artifacts and resistance. A moderate amount of electrode paste is required to attach electrodes, and excessive amounts of paste can cause electric bridges with other electrodes, which distorts the distribution of EEG. Furthermore, using too little paste also increases and causes error in measurements. The measurement environment is also important, and if the room is too hot or humid, it may cause problems such as sweating which may affect resistance and artifacts [44]. Therefore, EEG may generally demonstrate elevated or decreased findings depending on the measurement situation and resistance, thus it is necessary to interpret the results through relative power rather than absolute power in such cases. Accordingly, in this study, the group (d) subtype was thought to be relatively normal, which was confirmed by the relative power results. In addition, KARS and IVA+Plus results showed that group (d) performed better than other groups, which further supports the hypothesis (Fig 1E).

This study has several limitations. First, we failed to fully consider the IQ of the subjects, although we excluded subjects below an IQ of 70. It is known that EEG can vary depending on an individual's IQ, so this variable should be controlled for. Second, the number of participants among the three groups (ADHD, NOS, NT) was inconsistent, especially the number of NT [15]. Third, the study may have favored those more distracted and careless than ordinary children, because it targeted children who wanted to participate in ADHD research through posters. Fourth, although ADHD, NOS and NT each accounted for a major portion of Group(a), Group(b), Group(c), and Group(d), the proportion was still low because of the heterogenicity. A low proportion in the classification reduces the typicality and reliability of the results. Therefore, the distinction between ADHD, NOS and NT group is limited by the characteristics of QEEG. However, from the perspective that the NOS group also needed therapeutic intervention, the significance of this study lies in revealing the characteristics of QEEG in the ADHD group, including the NOS group, and its differences from the NT group. Lastly, we focused on the participants’ attention and chose not to focus on other symptoms, such as depression, that could affect the EEG results, for example, by causing elevated alpha waves. Although there are limitations, this study identified the QEEG characteristics that can be referenced by ADHD subtypes, which are thought to provide useful information for the diagnosis of ADHD. Future work on the QEEG characteristics of ADHD will further assist in the accuracy of diagnosis.

## 5. Conclusion

When diagnosing ADHD in clinical practice, QEEG is available as an auxiliary tool, providing additional information regarding the QEEG subtypes. An elevated delta power and decreased theta power, or an elevated theta power and decreased fast wave likely indicates a diagnosis of ADHD. On the other hand, if alpha waves are high, it is possible that attention-deficit symptoms may have been caused by childhood depression, or that a comorbidity such as childhood depression may be present rather than ADHD only. Finally, if absolute power is shown to be high overall, it is necessary to evaluate QEEG using relative power.

## Supporting information

S1 Fig Scatter plot of the K-ARS and IVA+Plus score between the ADHD, NOS and NT groups. Abbreviations: ADHD, Attention-Deficit Hyperactivity Disorder; NT, Neurotypical; NOS, ADHD Not Otherwise Specified; K-ARS, Korean ADHD rating scale, IVA+Plus, Integrated visual and auditory test; H/I, Hyperactivity/Impulsivity; Inatt., Inattention.
(TIF)

## Abbreviations

ADHD: Attention Deficit Hyperactivity Disorder
ANOVA: analysis of variance
CPT: Continuous Performance Test
DC: direct current
DISC-IV: Diagnostic Interview Schedule for Children Version IV
DSM-5: Diagnostic and Statistical Manual of Mental Disorders-5
f-MRI: functional magnetic resonance imaging
PCR: polymerase chain reaction
ICA: independent component analysis
IRB: Institutional Review Board
IVA CPT: Intermediate Visual and Auditory Continuous Performance Test
KARS: Korean ADHD rating scale
MRI: magnetic resonance imaging
NT: NeurotypicalNOS, Not Otherwise Specified
QEEG: quantitative electroencephalography
TBR: theta/beta ratio
