# Supplementary figures and images for: A novel quantitative electroencephalography subtype with high alpha power in ADHD: ADHD or misdiagnosed ADHD?
Source: PLoS One. 2020 Nov 17;15(11):e0242566. doi: 10.1371/journal.pone.0242566 (PMC7671485; doi:10.1371/journal.pone.0242566)

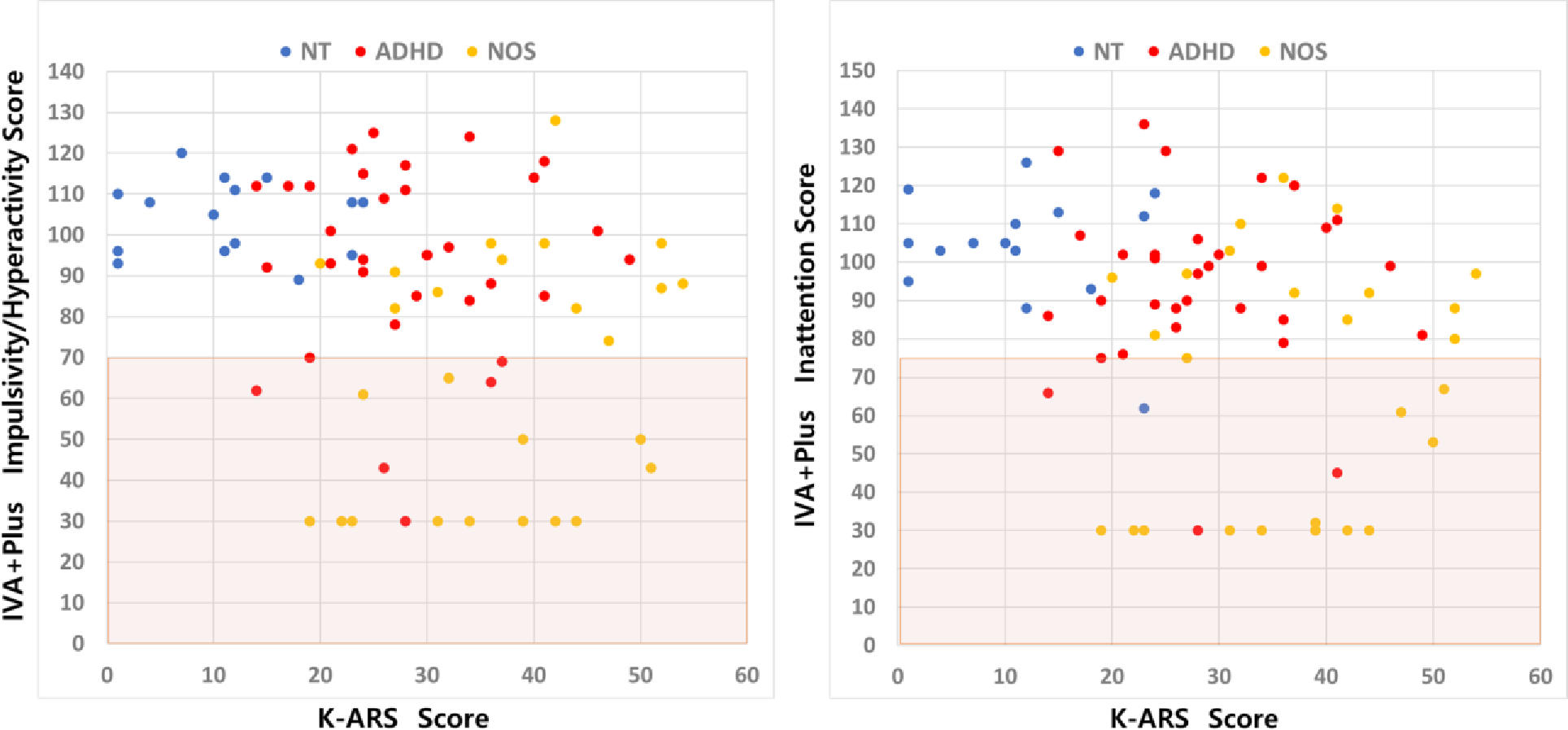

Supplement: S1 Fig — Abbreviations: ADHD, Attention-Deficit Hyperactivity Disorder; NT, Neurotypical; NOS, ADHD Not Otherwise Specified; K-ARS, Korean ADHD rating scale, IVA+Plus, Integrated visual and auditory test; H/I, Hyperactivity/Impulsivity; Inatt., Inattention. (TIF) [file pone.0242566.s001.tif]
